# Manufacturing and Shear Response Characterization of Carbon Nanofiber Modified CFRP Using the Out-of-Autoclave-Vacuum-Bag-Only Cure Process

**DOI:** 10.1155/2014/830295

**Published:** 2014-02-05

**Authors:** Erin E. McDonald, Landon F. Wallace, Gregory J. S. Hickman, Kuang-Ting Hsiao

**Affiliations:** Department of Mechanical Engineering, University of South Alabama, Mobile, AL 36688, USA

## Abstract

The interlaminar shear response is studied for carbon nanofiber (CNF) modified out-of-autoclave-vacuum-bag-only (OOA-VBO) carbon fiber reinforced plastic (CFRP). Commercial OOA-VBO prepregs were coated with a CNF modified epoxy solution and a control epoxy solution without CNF to make CNF modified samples and control samples, respectively. Tensile testingwas used to study the in-plane shear performance of [±45°]_4*s*_ composite laminates. Significant difference in failure modes between the control and CNF modified CFRPs was identified. The control samples experienced half-plane interlaminar delamination, whereas the CNF modified samples experienced a localized failure in the intralaminar region. Digital image correlation (DIC) surface strain results of the control sample showed no further surface strain increase along the delaminated section when the sample was further elongated prior to sample failure. On the other hand, the DIC results of the CNF modified sample showed that the surface strain increased relatively and uniformly across the CFRP as the sample was further elongated until sample failure. The failure mode evidence along with microscope pictures indicated that the CNF modification acted as a beneficial reinforcement inhibiting interlaminar delamination.

## 1. Introduction

Polymer matrix composites (PMC) have many applications in industry due to their high strength-to-weight ratios, corrosion resistance and lower susceptibility to fatigue [1–6]. They are replacing metals in many applications. For instance, in the case of aircraft structural applications, PMCs can possess similar mechanical properties to metals while weighing less and therefore reducing fuel cost and CO_2_ emissions [3]. A prevalent cause of failure of laminate composites is delamination in the interlaminar region where the composites are more susceptible to crack initiation and propagation [7, 8]. Nanofillers, such as carbon nanofibers and carbon nanotubes (CNF/CNT), are promising nanoconstituents to be introduced to composite matrices to increase the interlaminar toughness and enhance damage tolerance [4, 6]. Carbon nanofibers have excellent mechanical properties and are available at a lower cost than carbon nanotubes, making them a good candidate for strengthening composite matrices [1, 5].

The integrity of the structures is reliant not only on the materials in use but also on the method of manufacturing. Aerospace composite structures are typically cured by means of an autoclave [9]. A typical autoclave cycle involves inert gasses, such as nitrogen, and temperatures and pressures reaching 177°C and 375 kPa, respectively [10, 11]. Due to the cost and size limitations of autoclaves, the aerospace industry is investigating out-of-autoclave manufacturing methods [12, 13]. The out-of-autoclave-vacuum-bag-only (OOA-VBO) method is of interest because of the cost reduction, improved energy efficiency, and lesser limitations on the part size [9]. OOA-VBO prepregs are reliant on the vacuum channels in the prepreg to alleviate volatiles and excess resin from the composite during cure [12, 13].

The objective of this study was to investigate the changes in the interlaminar shear response of CFRP laminates manufactured from OOA-VBO prepregs coated with the compatible epoxy solutions with and without a 10wt% modification of oxidized carbon nanofibers. The samples used in this study had an epoxy solution and CNF/epoxy solution coated onto CYCOM 5320 T40/800 prepregs which made up the control and CNF modified samples, respectively. The prepregs were angle-orientated and stacked into a laminate composite in accordance with ASTM D 3518 and were cured using the OOA-VBO process. In addition to the interlaminar shear response of the samples, the tensile strain development during the testing process was studied using Digital Image Correlation (DIC), and optical microscopy was used in order to visually study the fracture surfaces of the samples. A significantly different failure mode caused by the CNF modification was concluded based on the evidences from the experiments. A micromechanics study was also conducted to explain the experimental finding.

## 2. Methods

### 2.1. Materials

The resin and hardener for the epoxy coating were Araldite MY 0510 and Aradur 9719-1, respectively, provided by Huntsman Advanced Materials America Inc. The Araldite MY 0510 was an aminophenol based liquid resin with a high glass transition temperature, low viscosity, and a water content less than 0.2% [14]. The Ardur 9719-1 was a micropulverized 3.3′-diaminoliphenyl sulfone powder with a mean particle size less than 60 *μ*m and water content less than 1% [15]. The oxidized carbon nanofibers used for the modification were PR-24 XT-LHT from Pyrograf Products, Inc. The average CNF particle diameter is 100 nm with carbon fiber composition at least 98% [16]. The carbon fiber prepregs were unidirectional CYCOM 5320 T40/800 supplied by Cytec Engineered Materials. Nominal fiber areal weight is specified as 145 gsm and the nominal resin content is specified as 33wt% [17].

### 2.2. Manufacturing

**Figure 1:**
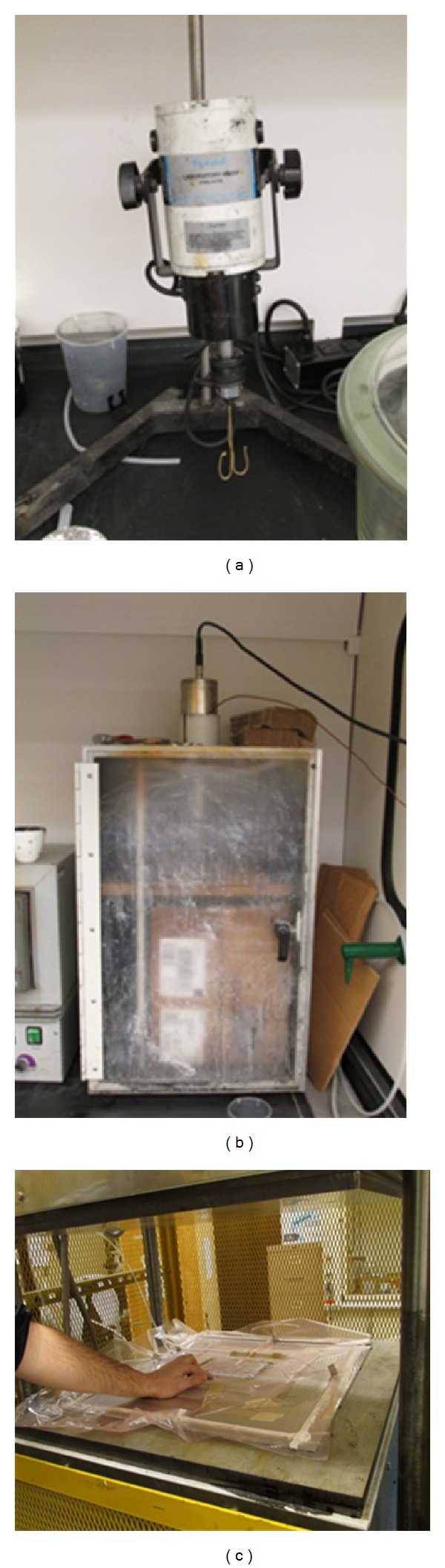
Mechanical mixer and sonicator ((a) and (b), resp.) used during resin and nanoresin preperation and vacuum bag system in open hotpress (c) for OOA-VBO cure.

The resin and hardener for the control samples were mixed using a ratio of 100 : 44 as per manufacturer specifications. The solution was mechanically mixed, using the Cole Parmer Stir-Pak laboratory mixer shown in Figure 1(a), and applied to the top surfaces of the CYCOM 5320 T40/800 prepregs using a roller. To prepare the CNF modified samples, 10wt% oxidized CNF was added and mixed to the resin under vacuum. 200–300wt% acetone was added to the solution for better dispersion. The solution was then sonicated for one hour using the Q700 model Qsonica sonicator shown in Figure 1(b). The hardener was added after sonication and the solution was applied similarly to the control samples. The coated prepregs were allowed to sit prior to stacking so that the acetone in the CNF modified solution could evaporate. The laminates for both control and CNF modified samples were stacked in a [±45°]_4*s*_ (16 plies) orientation as per ASTM D 3518/D 3518 M and were cured in accordance with the Cycom 5320 data sheet using the OOA-VBO setup in a closed hot press.  Figure 1(c) illustrates the vacuum bagged panel sitting in the open hot press prior to curing. The cure cycle began with a temperature of 121 ± 6°C for 1 hour and concluded at a temperature of 177 ± 6°C for 2 hours. The temperature ramp rate was 0.6–2.8°C/min.

### 2.3. Test Methods

**Figure 2:**
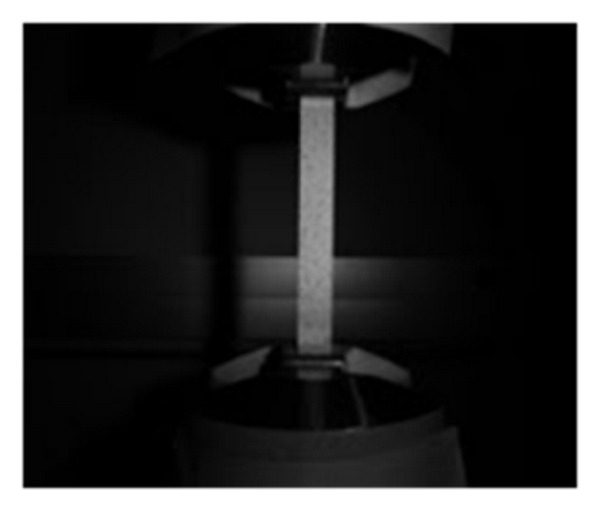
Tensile sample with DIC speckle pattern setup in the MTS grips.

The laminate CFRP samples were tested for interlaminar shear response in accordance with ASTM D 3518/D 3518M-94 (reapproved 2001),* “Standard Test Method for In-Plane Shear Response of Polymer Matrix Composite Materials by Tensile Test of a *±45°* Laminate.” *This method specifies the use of a uniaxial tension test of a ±45° laminate composite using specifications from ASTM D 3039/D 3039 M-00(2006), *“Standard Test Method for Tensile Properties of Polymer Matrix Composite Materials.” *The samples were cut from laminate composite panels into rectangular tensile test samples with an average cross-sectional area of 59.705 mm^2^ and an average length of 205.175 mm. The tabs for the samples were 25 mm square 0°/90° E-glass fiber reinforced polymer matrix tabs. An 810 Material Test System (MTS) and MTS 647 Hydraulic Wedge Grips were used to conduct the tensile tests. The head displacement rate was constant at 2 mm/min. The stress data from the MTS was used in interlaminar shear response analysis. Digital Image Correlation (DIC) was used to collect surface strain data as opposed to using strain gages or an extensometer. The samples, prior to tensile testing, were spray painted white with a black speckle pattern for DIC use. Two-dimensional images were taken during testing every 500 ms using a 105 mm F2.8 DG MACRO Sigma lens for Nikon AF. The raw strain data was collected using VIC-Snap 2D, Correlated Images software, for 2D analyses. The initial image before force was applied was used as a reference image to the initial speckle pattern prior to loading.  Figure 2 displays a speckle painted sample loaded in the MTS grips just before tensile testing.

## 3. Results

**Figure 3:**
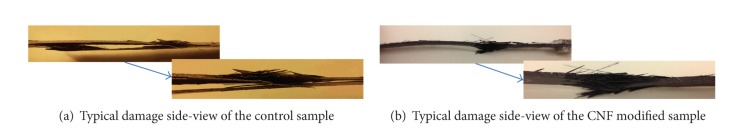
Enhanced side-views show delamination in control samples (a) and localized failure in CNF modified samples (b).

Figure 3 displays the side-views of typical control and CNF modified samples. The control samples, represented by Figure 3(a), experienced a half-plane delamination propagating along the lengths of the samples, leading to the separation of the prepreg plies. Fiber failure occurred in the ±45° directions along the orientation of the plies. The CNF modified samples, on the other hand, represented by Figure 3(b), experienced a localized failure, where fiber failure also occurred in the ±45° directions. No half-plane delamination or damage propagation was found in any CNF modified sample. The CNF modified epoxy coating was observed to effectively inhibit the delamination of the laminates and restrain the damage propagation.

**Figure 4:**
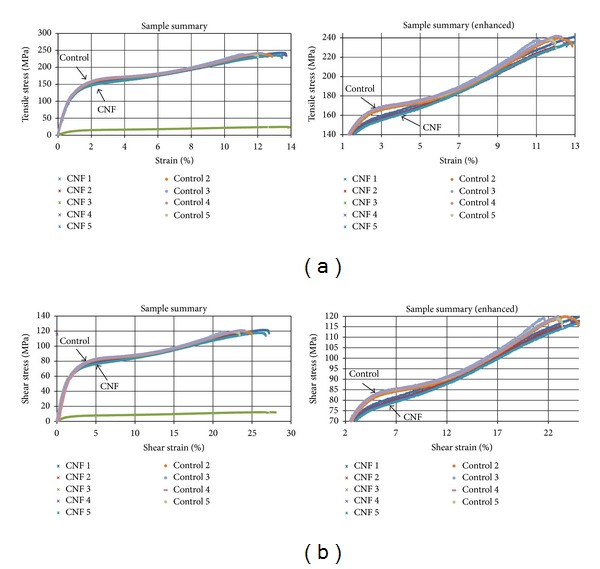
Tensile stress-tensile strain (a) and shear stress-shear strain (b) graphs for all tested samples. The enhanced graph shows the difference between the control and CNF modified samples just after yielding. The single data curve outlier in both graphs is from the CNF 3 sample. This sample experienced a testing flaw and was not included in the calculations for Table 1.

The graphs in Figure 4 represent the tensile stress-tensile strain and shear stress-shear strain data for the samples where strain is represented as a percentage. The curves of the control and CNF modified samples exhibit similar behavior in the elastic region before the ultimate tensile strength was reached. The difference displayed in the curves occurred just after the samples began to yield. The control samples transition into the plastic region differently from the CNF modified samples due to, and marking the initiation of, carbon fiber rotation and delamination in the interlaminar region. Delamination in the interlaminar region of the control samples continues until the curves for both types of samples overlap again. Further damage experienced by the control samples occurred in the intralaminar region as fibers begin to experience pulling and fiber scissoring. The damage to the CNF modified samples was primarily observed in the intralaminar region implying the CNF modified matrix toughened the interlaminar region and better prevented carbon fiber rotation from scissoring. Based on Section 13.1 of ASTM D 3518, such difference in the stress-strain curves indicates an increase in matrix ductility as a result of the addition of a 10wt% CNF matrix. The calculated shear properties in Table 1 support the similarities observed between the curves aside from failure behavior.

**Figure 5:**
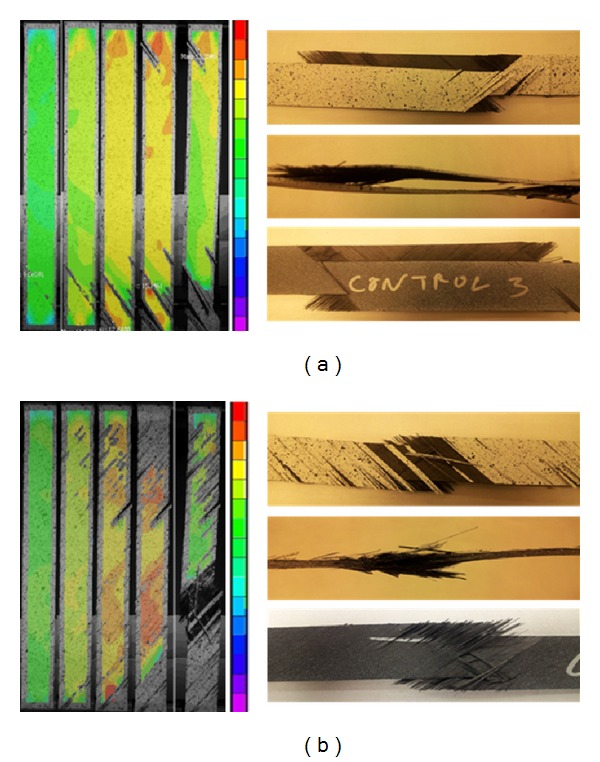
DIC images for a representative control sample (a) and for a representative CNF modified sample (b). The screen shots represent the samples as they begin to experience the color coordinated strain levels. The colors as described by the color bars to the right of the images represent strain concentrations on the front of the samples. The pictures of the fractured samples are also shown in the front-view, side-view, and back-view to be compared with the DIC images.

The images in Figure 5 represent the surface strain evolution of a control sample (Figure 5(a)) and a CNF sample (Figure 5(b)) as captured by the DIC system. The scale to the right of each sample represents the tensile strain percentage in the vertical direction. The scale percentage values vary slightly between samples but the typical values range from 0% at the bottom of the scale to 14–16%. The DIC measured strain varies from the calculated maximum average shear strain calculated in Table 1 by roughly 10%. This is due to the DIC only measuring surface strain on the front face of the sample. In other words, the fibers can experience rotation, pull out, or breakage on the back side of the sample and in the middle of the sample and not be recorded by the camera. On the other hand, these images allow analysis of front face surface strain concentrations throughout the tensile loading. The control sample experienced surface damage at the bottom of the sample initially. Greater strain percentages began to appear at the top of the sample as the interlaminar damage progressed from the bottom to the middle. During this evolution, the initial cross-sectional failure occurred at the bottom of the sample and the interlaminar crack progressed to the top of the sample. It is suspected that greater local strain occurred at locations where more local stress was received due to the damage development on the sample and the fact that some parts of the sample cannot effectively share the tensile load anymore. Surface damage occurred on the CNF sample in a more uniform pattern than the control sample. In fact, it is intriguing to find that the initial surface damage sites were away from the location of final failure in Figure 5(b). The final frame of the CNF sample showed the location of the localized failure. In the frames just before failure, high strain percentages were measured leading up to the localized cross-sectional failure. While these analyses are specific to the images in Figure 5, the behavior described for both types of samples can be applied to all samples in the DIC study. The grey areas in the final DIC images in Figures 5(a) and 5(b) are due to the samples changing position and becoming out of focus during fracture [19].

**Figure 6:**
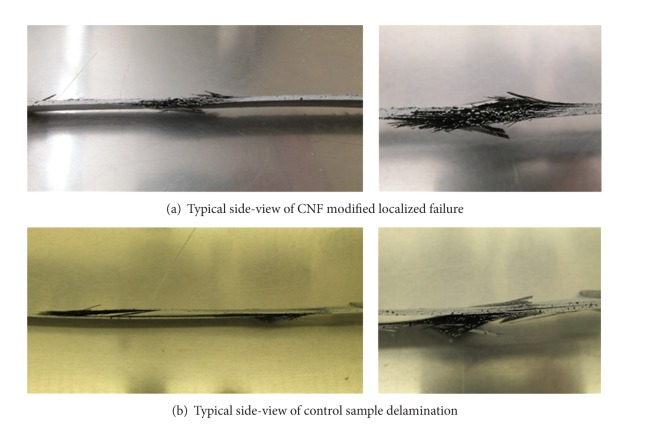
Representative close-up images of side surface damage to the CNF modified samples (a) and of the control images (b). The CNF modified samples show multiple layer damage representing localized failure occurring through the cross-section of the samples primarily. The control samples show cross-sectional initial failure on a surface, followed by delamination through the midplane and final cross-sectional failure on the opposite surface.

Figure 6 shows a representative close up of cracks for both types of samples in such a way as to notice the differences near the surface damage locations. The CNF modified samples (Figure 6(a)) had only the cross-sectional and localized failure that consisted of the uniform separation of all the layers and fiber separation from the matrix. This supports the previous analyses that the CNF addition lessened interlaminar fiber-scissoring and therefore inhibited delamination. The control samples, with an unreinforced matrix, experienced interlaminar crack propagation axially along the mid-plane of the samples.  Figure 6(b) shows that the crack extends further than the final cross-sectional failure, which supports the data in Figure 4. As previously indicated, the control samples' stress data indicates a behavioral difference from the CNF modified samples when entering the plastic region of the curves.  Figure 6(b) provides support that the hump in the graph could represent the allowance of fiber scissoring and delamination along the length of the sample. The graphs overlap again when the lengthwise crack propagation ends and cross-sectional failure continues. The failure through the cross-section at ultimate tensile strength, which is typically dominated by the carbon fibers, agrees with the failure of the nanomodified samples. Therefore, as seen and stated in Figure 4 and Table 1, respectively, the ultimate tensile strength and shear response values of the control and CNF samples are not significantly different. The behavior of the control and CNF samples during loading after the elastic range, however, are significantly different.

The modulus of elasticity (Table 1) was calculated with a strain range of 0–0.0125. The shear chord modulus of elasticity of the CNF modified CFRP is less than 1% different from the control CFRP. Other shear response properties do not show a significant difference with the 10wt% CNF modification. The shear strain data was calculated from DIC data and was reported as a percentage. The percentages seemed high with values of 25.65% and 23.48% for the CNF modified and control samples, respectively. The values were verified by comparing DIC and MTS data. The data from both sources agreed and were, therefore, considered to be accurate. The high percentages may have occurred because of the fiber pulling and rotation prior to failure.

**Figure 7:**
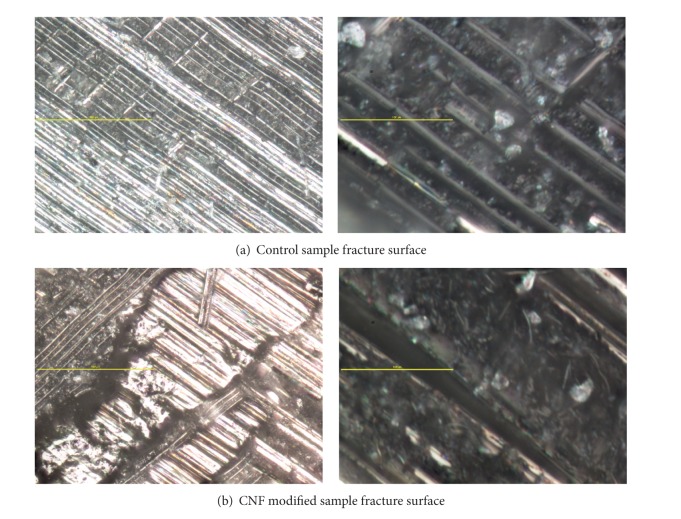
Surface morphology using Nikon Eclipse LV150 optical microscope.  Figure 7(a) represents the control sample morphology at 400x and 2000x from left to right, respectively.  Figure 7(b) represents the CNF modified sample morphology at 400x and 2000x from left to right, respectively. The scales for the 400x images measure 500 micrometers and the scales for the 2000x images measure 100 micrometers.

Typical fracture surfaces are shown in Figure 7. The prepreg fibers were unidirectional and depths depict prepreg layers. The microscope images were taken close to the center of the sample, away from the side edges.  Figure 7(a) represents the control samples where the right image has a 2000x magnification and the left image is a 400x magnification zoom-out of the 2000x location surface.  Figure 7(b) represents the CNF modified samples with the same image specifications. The control sample shows two to three layers of fibers supporting axial damage and delamination. The CNF modified 2000x sample surface shows the dispersed carbon nanofibers in the nanoresin. The region shown is possibly at an interface due to the presence of both carbon fibers and indentations where carbon fibers were pulled away from the surface. Due to apparent matrix toughening and the presence of pulled fibers, the fiber pulling was determined to be from the intralaminar region instead of the interlaminar region of the samples. The 400x CNF modified sample surface image supports more transversely directed failure due to the amount of visible layers of prepregs.

To better understand the micromechanics associated with the different failure modes between the control CFRP sample and the CNF modified CFRP sample, an analytical modeling approach is used to compare the shear strengths of the pure epoxy, the CNF modified epoxy, and the T-40 carbon fiber/epoxy lamina. The shear strength of the pure epoxy is equal to half of its tensile strength, which is a basic material property. The shear strength of randomly aligned CNF modified epoxy can be predicted with a micromechanics model derived by modifying the Cox model [20] (see the Appendix). The shear strength in the T-40 carbon fiber/epoxy lamina, however, is too complex to be modeled by an analytical solution and is assumed to be close to and slightly higher than the shear strength of pure epoxy. The approximated material properties of the standard high performance epoxy and CNF (vapor grown, average length ~100 *μ*m and average diameter ~0.2 *μ*m) are obtained from [16–18] and listed in Table 2. Based on the density data in Table 2, the 10wt% CNF concentration is equivalent to 6.9% CNF volume fraction in the CNF/epoxy system.  Table 3 shows the predicted shear moduli and shear strengths of the pure epoxy and the CNF modified epoxy. The micromechanics model to predict the shear properties of the CNF modified epoxy is detailed in the Appendix.

Theoretically, the shear failure initiates at a region with the lowest material shear strength. According to Table 3 and the assumption that the shear strength of the T-40 carbon fiber/epoxy CFRP lamina is close to and slightly higher than the shear strength of the pure epoxy, it is very likely that the shear strength of the CNF modified epoxy is the highest one among all and is followed by the CFRP lamina and the pure epoxy. Therefore, the shear failure of the control CFRP sample is predicted to start at the interlaminar region, which is filled with pure epoxy. On the other hand, the shear failure of the CNF modified CFRP is predicted to begin at the T-40 carbon fiber/epoxy lamina, due to the increased shear strength of the interlaminar region, which is filled with randomly orientated CNF modified epoxy. This analytical study predicts the two distinct failure initiation modes between the control CFRP and the CNF modified CFRP that are comparable to the experimental observation.

## 4. Conclusion

**Figure 8:**
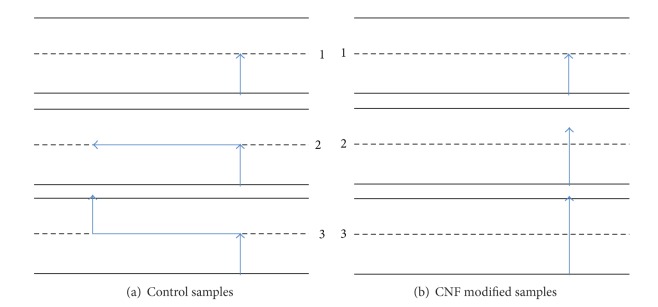
Damage development, as concluded by the presented evidence, of both the control samples (a) and the CNF modified samples (b). The development is presented in three steps. The control sample steps include: initial localized cross-sectional failure, delamination along the mid-plane, and final localized cross-sectional failure. The CNF modified samples experience only localized failure.

The interlaminar shear response was studied for a 10wt% CNF modified epoxy matrix of a carbon fiber laminate composite. The calculated values of ultimate tensile strength and maximum shear stress and shear strain showed no significant difference with the addition of 10wt% CNF and the modulus chord of elasticity showed a less than 1% difference. The failure modes of the samples, on the other hand, were found to be significantly different as summarized in Figure 8 with the three concluded steps in damage development for the control samples (Figure 8(a)) and for the CNF modified samples (Figure 8(b)).

The control samples experienced interlaminar delamination propagation along mid-plane following the sample length (Figure 8(a)) and fiber scissoring immediately after yielding. The CNF modified samples conversely experienced a localized cross-sectional failure (Figure 8(b)) and less carbon fiber rotation. The fiber pullout experienced by the CNF modified samples was determined to be in the intralaminar region instead of the interlaminar region due to matrix toughening from the nanofiller. The microscope images allow visuals of the nanoresin toward the interfacial surfaces as a result of transverse fracture. It is concluded that the 10wt% CNF addition to the epoxy matrix solution successfully inhibited interlaminar delamination and restrained the damage propagation. A micromechanics study suggested this delamination prevention may be due to the shear strength improvement at the interlaminar region caused by the addition of CNF. Future work should introduce CNF into the intralaminar sites of the prepreg system to study further possible improvement in intralaminar performance.

## Figures and Tables

**Table 1:** Average interlaminar shear response with standard deviations and coefficients of variation.

	Max shear stress (MPa)	Max shear strain (%)	Ultimate tensile strength (MPa)	Shear chord modulus of elasticity (MPa)
CNF modified samples				
Average	118.96	25.65	237.87	4031.31
stdev	1.80	2.38	3.44	66.70
CV	1.51	9.28	1.44	1.65

Control samples				
Average	119.76	23.48	239.52	4131.84
stdev	0.81	1.23	1.63	80.94
CV	0.68	5.24	0.68	1.96

**Table 2:** The materials properties [16–18] used in the micromechanics model.

Symbol	Definition	Value
*E*_resin_	Epoxy resin modulus	3.854 GPa
*E*_CNF_	CNF modulus	600 GPa
*S*_resin_	Epoxy resin tensile strength	0.141 GPa
*S*_CNF_	CNF tensile strength	7.0 GPa
*λ*_resin_	Poison's ratio of epoxy resin	0.265
*λ*_CNF_	Poison's ratio CNF (assumed to be the same as a typical carbon fiber)	0.2
*v*_CNF_	CNF volume fraction	0.069
*v*_*m*_	Matrix volume fraction	1 − *v*_CNF_
*ρ*_CNF_	CNF density	1.8 g/cm^3^
*ρ*_resin_	Epoxy resin density	1.2 g/cm^3^
*l*_CNF_	Length of CNF	100 *μ*m
*d*_CNF_	Diameter of CNF	0.2 *μ*m

**Table 3:** Predicted shear moduli and failure strengths of the pure epoxy and CNF modified epoxy.

System	Shear modulus (GPa)	Shear strength (MPa)
Pure epoxy	1.523	70.3
Randomly oriented CNF/epoxy	4.178	191.4

## Appendix

This section provides the analytical modeling of the shear moduli and shear strengths of pure epoxy and randomly oriented CNF modified epoxy.

For the epoxy matrix, the shear modulus can be calculated as

(A.1) $$\begin{array}{c} G_{m}=\frac{E_{\text{resin}}}{2\left(1+\lambda_{\text{resin}}\right)}. \end{array}$$

The max shear strain of resin, *γ*_max⁡,*m*_, is calculated from the maximum tensile strain of the resin and is given as

(A.2) $$\begin{array}{c} \gamma_{\max,m}=\left(1+\lambda_{\text{resin}}\right)\frac{S_{\text{resin}}}{E_{\text{resin}}}. \end{array}$$

The shear strength of the resin can be calculated as

(A.3) $$\begin{array}{c} \tau_{\max,m}=G_{m}\gamma_{\max,m}=\frac{S_{\text{resin}}}{2}. \end{array}$$

To model the CNF modified epoxy system, a micromechanics approach is used. First, the shear modulus of the randomly orientated CNF modified epoxy is approximated by modifying the Cox model [20]. The Cox model was originally developed for predicting the elastic properties of fibrous materials that consisted either 2D or 3D randomly aligned fibers without matrix. For the 3D randomly aligned CNF modified epoxy, the matrix's contribution can be superimposed with the contribution predicted from Cox model. Thus the tensile modulus can be approximated as

(A.4) $$\begin{array}{c} E_{\text{random}\;\text{CNF-resin}}=\frac{1}{6}E_{\text{CNF}}v_{\text{CNF}}+E_{\text{resin}}v_{m}. \end{array}$$

Similarly, the shear modulus of the 3D randomly aligned CNF modified epoxy can be approximated as

(A.5) $$\begin{array}{c} G_{\text{random CNF-resin}}=\frac{1}{15}E_{\text{CNF}}v_{\text{CNF}}+\frac{E_{\text{resin}}}{2\left(1+\lambda_{\text{resin}}\right)}\;v_{m}. \end{array}$$

And the Poison's ratio is given as

(A.6) $$\begin{array}{c} \lambda_{\text{random CNF-resin}}=\frac{E_{\text{random CNF-resin}}}{2G_{\text{random CNF-resin}}}-1. \end{array}$$

The maximum tensile strain of the randomly oriented CNF modified epoxy is assumed be same as the resin's maximum tensile strain. Therefore, the maximum shear strain of the randomly oriented CNF-resin nanocomposite can be approximated as

(A.7) $$\begin{array}{c} \gamma_{\max,\text{random}\;\text{CNF-resin}}=\left(1+\lambda_{\text{random CNF-resin}}\right)\frac{S_{\text{resin}}}{E_{\text{resin}}}. \end{array}$$

Hence, the shear strength of the randomly orientated CNF modified epoxy can be approximated as

(A.8) $$\begin{array}{c} \tau_{\text{max,random CNF-resin}}=\gamma_{\text{max,random CNF-resin }}G_{\text{random CNF-resin}}. \end{array}$$

Note that, this modified Cox model can possibly overestimate the shear strength of the randomly oriented CNF modified epoxy since it is unable to correspond to the possible scenario that the CNFs can be broken into various lengths and carry less load inside the CNF/epoxy nanocomposite before the epoxy matrix reaches its maximum tensile strain.
